# Stress adaptation in older adults with and without cognitive impairment: an fMRI pattern-based similarity analysis

**DOI:** 10.18632/aging.102204

**Published:** 2019-09-03

**Authors:** Xixi Wang, Kathi L. Heffner, Mia Anthony, Feng Lin

**Affiliations:** 1 Department of Biomedical Engineering, University of Rochester, Rochester, NY 14642, USA; 2 School of Nursing, University of Rochester, Rochester, NY 14642, USA; 3 Department of Psychiatry, University of Rochester Medical Center, Rochester, NY 14642, USA; 4 Department of Medicine, Division of Geriatrics and Aging, University of Rochester Medical Center, Rochester, NY 14642, USA; 5 Department of Brain and Cognitive Sciences, University of Rochester, Rochester, NY 14642, USA; 6 Department of Neuroscience, University of Rochester Medical Center, Rochester, NY 14642, USA; 7 Department of Neurology, University of Rochester Medical Center, Rochester, NY 14642, USA

**Keywords:** stress adaptation, amnestic mild cognitive impairment, functional magnetic resonance imaging, pattern analysis

## Abstract

Background: The capacity to adapt to environmental stressors is essential for older adults’ health and well-being. It is unclear how cognitive impairment may disrupt the capacity. Here we examined the relationship between self-perceptions of stress and the neurobiological response to a laboratory model of stress adaptation in amnestic mild cognitive impairment (aMCI), a group at high risk for dementia.

Results: aMCI group and cognitively healthy controls did not differ in neurobiological acute stress recovery (indexed by similarity in neural patterns at baseline and after recovery from cognitive challenges). However, compared to controls, aMCI group had significantly lower scores on PSS-PW. Notably, higher PSS-PW was associated with greater acute neural recovery in controls, but not aMCI.

Methods: We assessed self-perceptions of stress adaptation with the Perceived Stress Scale subscales, measuring perceived helplessness (i.e., negatively worded items, PSS-NW) and self-efficacy (i.e., positively worded items, PSS-PW) in response to stress. At a subsequent laboratory fMRI visit, we indexed neurobiological stress adaptation by assessing and comparing functional network connectivity at baseline and immediately following, and after recovery from, cognitive challenges.

Conclusions: Studying stress adaptation in aMCI may shed light on pathways that contribute to the onset and progress of cognitive deterioration in aging.

## INTRODUCTION

Cumulative or repeated maladaptive physiological and behavioral responses to stressful experiences can compromise one’s physical and mental health [1, 2]. Conversely, an individual’s capacity to respond flexibly and adaptively to environmental stressors can be health protective [3]. Neurobiological evidence suggests key neural pathways that play a role in adaptation to changing demands and stressors [4]. Among older adults, there is evidence that even subtle aging-related cognitive deficits reduce one’s capacity for stress adaptation [5]. Thus, cognitive deficits, along with additional aging-related changes in physiological regulation, may render older adults particularly vulnerable to the effects of maladaptive stress responses on health [5–9]. The significance of this evidence for older adults with cognitive impairment is unknown. Yet, older adults with cognitive impairments can face ongoing demands and stressors related their daily living activities (e.g., problem solving, managing finances, remembering names, etc.). Maladaptive stress responses to these cognitive demands may exacerbate poor health, including further cognitive decline in these older adults at risk for dementia, given known effects of stress on accelerated cognitive aging [10, 11]. In the current study, we aimed to understand the role of cognitive impairment in subjective and neural markers of stress adaptation.

One important mechanism contributing to stress adaptation is the maintenance of cerebral blood oxygen tension [12]. As such, the neural profile of stress adaptation has received more recent attention. Activity of the hippocampus and prefrontal cortex (PFC) plays a primary role in the stress response [13]. Dynamic changes occur in these regions and associated neural networks (e.g., executive control network, default mode network) [14–17], which regulate homeostatic processes as well as behavioral responses to changing or demanding environmental demands [18, 19]. Pathophysiological aging can lead to reorganization or dysfunction in these brain regions or relevant neural networks [20]. It is unclear whether such neural reorganization or dysfunction, along with consequential cognitive decline, affects stress adaptation.

Theoretical models of stress suggest that stress arises when perceived demands outweigh perceived resources to cope [21, 22]. Aging [23] or behavioral symptoms in old age (e.g., psychosis [24]) can affect stress and coping processes, and more recent evidence suggests that perceived capacity to cope with stressors, rather than overall perceptions of stress, may be uniquely predictive of incident amnestic mild cognitive impairment [25, 26]. The implications of AD-related cognitive impairment and pathophysiology for stress and coping perceptions and neural networks integral to stress adaptation are unclear. To fully understand the implications of cognitive impairment for stress adaptation, it will be necessary to characterize the interplay of perceived stress and coping and stress-regulating neural pathways. Alzheimer’s disease (AD), the most common neurodegenerative disorder in aging populations, provides a testable context for understanding how cognitive decline and neurodegeneration may affect stress perceptions and concomitant neural pathways involved in stress adaptation. Evaluating both general perceptions of stress and neural responses to cognitive challenges in those with amnestic mild cognitive impairment (aMCI) and in cognitively healthy older counterparts (HC) may help clarify how AD neurodegeneration influences multiple, integrated contributions to stress adaptation.

In the current study, the relationship between perceived stress and neural patterns of stress adaptation to cognitive challenges were examined and compared between aMCI and HC groups. Neural recovery – or more specifically, how fast individuals’ brain function return to baseline following an environmental challenge – can provide an index of an individual’s capacity for stress adaptation [27, 28]. Notably, cumulative studies have revealed differences in default neural networks between aMCI and HC [29–31]. In this study, patterns of neural network recovery following demanding cognitive challenges were examined within these networks. We suspected that certain “functional network connectivity” [32] within default neural networks that are vulnerable to AD-associated neurodegeneration [29] would consequently impact stress adaptation, reflected in less neural recovery following an acute stressor. Here we were interested in the connectivity among networks instead of between regions because stress regulation and adaptation involves multiple inter-network connectivity seeded in the hippocampus and PFC [33]. Given this reliance on inter-network (rather than region-specific network) connectivity, studying “functional network connectivity” should provide a fuller picture for understanding stress adaptation in the context of AD, compared to region-based functional connectivity. We implemented pattern-based similarity analysis to assess acute neural recovery from the cognitive challenges. Recovery is indexed by the overall degree of correspondence between two sets of neural representations, namely the resting-state functional network connectivity following challenge compared to baseline [34]. Specifically, more adaptive stress recovery would be indexed by higher similarity in the neural patterns between stages. Here, we tested the hypothesis that, compared to older adults without cognitive impairment, older adults with aMCI would show indications of more compromised stress adaptation capacity, evidenced by more global perceptions of stress, particularly reduced coping capacity, and poorer neural recovery from demanding cognitive challenges. The associations between global perceived stress and neural recovery within groups were explored to further characterize the psychoneurobiological concomitants of stress adaptation (or maladaptation) within the context of AD-related cognitive impairment.

## RESULTS

### Group independent component analysis (ICA)

**Figure 1:**
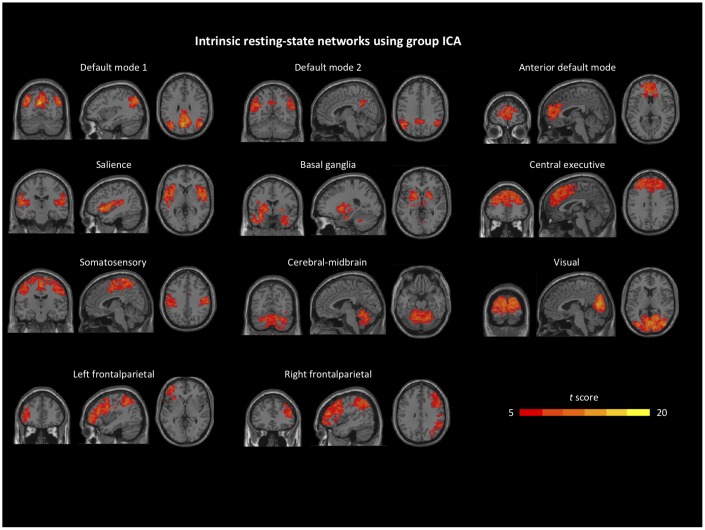
**Identified intrinsic resting-state network components from group independent component analysis.** Details can be found in the *Group ICA* section.

From the 20 independent components, we identified 11 resting-state network components (corrected FDR *p* < 0.001, cluster size > 100 for one-sample *t*-tests) based on previous findings [29], including the default mode network (DMN), anterior default mode network (aDMN), salience network (SAL), basal ganglia (BG), central executive network (CEN), frontalparietal network (FPN), somatosensory network (SSN), visual network (VIS), and cerebellum-midbrain network. We then performed two-sample *t*-tests on each resting-state network contrasting the two groups, back reconstructed independent component patterns at baseline (Figure 1).

### Sample characteristics

Sample characteristics for the HC and aMCI groups are presented in Table 1.

**Table 1:** Sample characteristics.

	**HC (n = 18)**	**aMCI (n = 17)**	**t or χ^2^ test, df (p)**
Age, Mean (SD)	71.44 (9.93)	73.94 (10.70)	-0.72, 33 (.48)
Male, N (%)	7 (38.9)	8 (47.1)	0.24, 1 (.74)
Education, Mean (SD)	16.06 (2.24)	15.24 (2.89)	0.80, 33 (.35)
MOCA, Mean (SD)	26.11 (2.72)	24.12 (2.62)	**2.21, 33 (.034)**
GDS, Mean (SD)	1.22 (1.96)	2.71 (2.52)	-1.95, 33 (.059)
Stroop_IIVRT, Mean (SD)	0.29 (0.07)	0.37 (0.10)	**-2.83, 33 (.008)**
NBack_IIVRT, Mean (SD)	0.30 (0.06)	0.41 (0.10)	**-4.05, 33 (.001)**
Whole brain VBM, Mean (SD)	577.23 (62.44)	536.86 (56.27)	2.01, 33 (.053)
PSS-PW, Mean (SD)^a^	1.24 (0.12)	1.08 (0.30)	**2.11, 33 (.043)**
PSS-NW, Mean (SD)^a^	-0.20 (0.67)	-0.06 (0.76)	-0.60, 33 (.55)
Neural recovery: baseline/recovery^a^	0.59 (0.17)	0.62 (0.17)	-0.49, 33 (.63)
Neural recovery: change^a^	0.47 (0.24)	0.53 (0.21)	-0.73, 33 (.47)

MOCA: Montreal Cognitive Assessment; GDS: Geriatric Depression Scale; Stroop_IIVRT: intra-individual difference in reaction time for Stroop task; NBack_IIVRT: intra-individual difference in reaction time for Dual 1-back task; VBM: Voxel-Based Morphometry; PSS: Perceived Stress Scale; PSS-PW: perceived self-efficacy; PSS-NW: perceived helplessness; SD: standard deviation. Note: ^a^a total of 35 participants have completed PSS data were included in the statistical analysis. PSS data were natural log transformed.

### Group comparison for Perceived Stress Scale (PSS)

The HC group had significantly higher positively worded (PSS-PW) scores compared to the aMCI group (t_(33)_ = 2.11, *p* = .043) but similar negatively worded (PSS-NW) scores (t_(33)_ = -0.60, *p* = .55).

For all subjects, higher PSS-PW, but not PSS-NW, scores were also significantly correlated with better Montreal Cognitive Assessment (MoCA) scores (r_(35)_ = 0.35, *p* = .039).

### Group comparison for neural recovery

No significant group differences were observed: t_(33)_ = -0.49, *p* = .63 for *neural recovery: baseline/recovery*; t_(33)_ = -0.73, *p* = .47 for *neural recovery: change*.

### Associations of PSS subscales with neural recovery

Neither PSS-PW nor PSS-NW was correlated to neural recovery indices for the entire sample (all *p* > .30).

**Figure 2:**
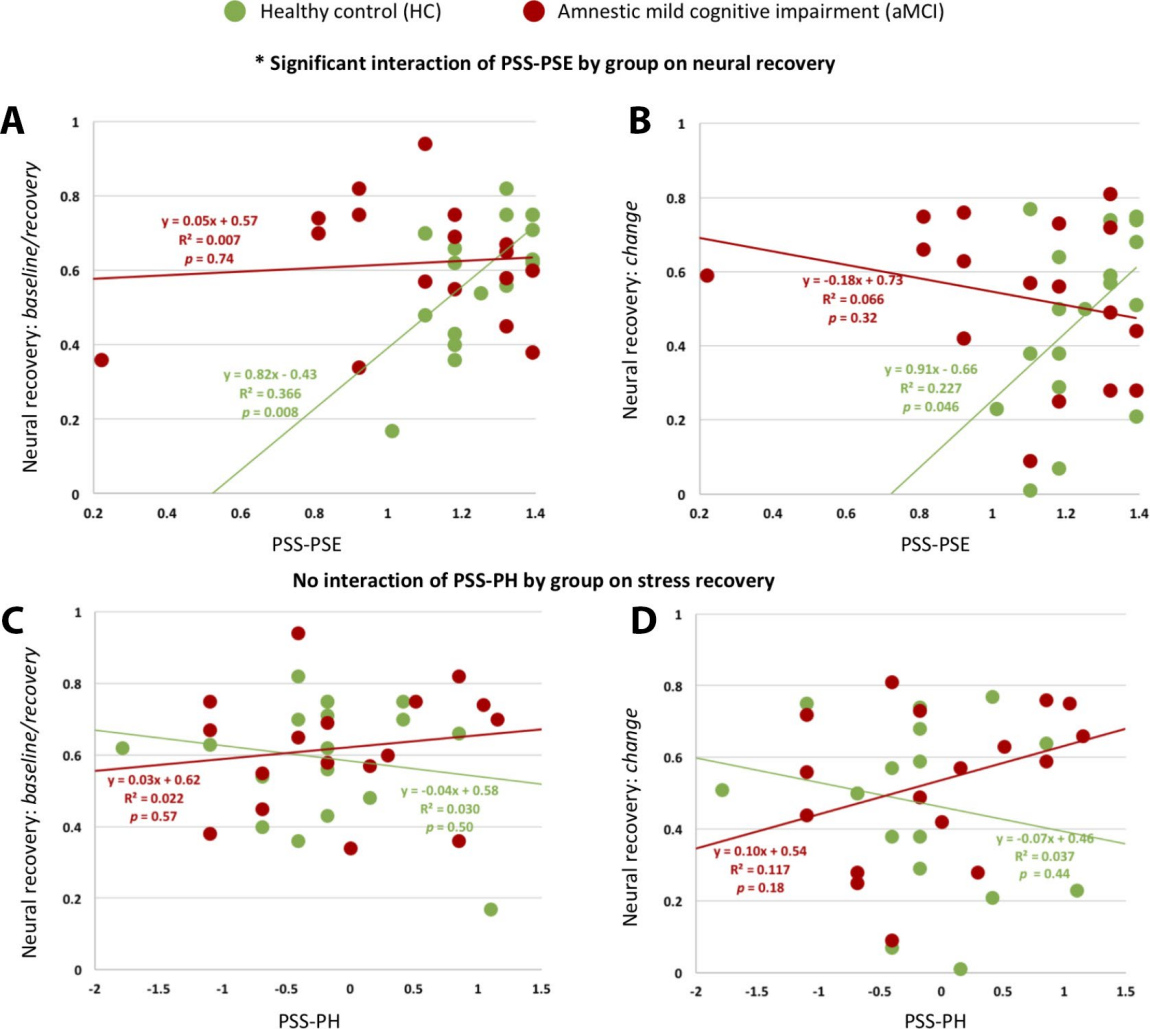
**Associations of PSS subscales with neural recovery.** There were significant interaction effects of PSS-PW by group on neural recovery: *baseline/recovery*. (**A**) and neural recovery: *change* (**B**). There was no interaction effect of PSS-NW by group on neural recovery: *baseline/recovery* (**C**) or neural recovery: *change* (**D**).

Applying Generalized Linear Models (GLM) analysis, there was a significant interaction effect of PSS-PW by group on neural recovery (B(SE) = -0.78 (0.31), Wald $\chi_{\left(1\right)}^{2}=6.29$, *p* = .012 for *neural recovery: baseline/ recovery*; B(SE) = -1.10 (0.43), Wald $\chi_{\left(1\right)}^{2}=6.57$, *p* = .010 for *neural recovery: change*) (Figure 2A, 2B). Of note, controlling for depression, age, global cognition, and whole brain voxel-based morphometry (VBM) did not affect the significance of these results. For the post-hoc analysis, higher PSS-PW scores significantly correlated with neural recovery in the HC group (r_(18)_ = 0.61, *p* = .008 for *neural recovery: baseline/recovery*; r_(18)_ = 0.48, *p* = .046 for *neural recovery: change*.) No correlations were observed between PSS-PW and neural recovery in the aMCI group (Figure 2C, 2D).

There was no interaction effect of PSS-NW by group on either neural recovery measurement.

## DISCUSSION

In the current study, we examined the role of AD risk in older adults’ capacity for stress adaptation, focusing global stress perceptions and neural recovery from an acute challenge. The aMCI group had significantly lower scores on the positively worded subscale of the PSS than HC, suggesting lower perceived capacity to cope with stress. aMCI and HC groups had similar scores on the negatively worded PSS subscale, suggesting no differences in perceived stress or helplessness. The two groups showed similar neural patterns of acute stress recovery. When exploring links between stress perceptions and neural recovery, only among the HC group was more perceived coping capacity associated with greater neural recovery from cognitive challenges. Coping perceptions and neural recovery were not associated among older adults with aMCI, suggesting that links between stress and coping-related perceptions and neural function may be weakened during the development of AD.

Consistent with previous studies [35, 36], perceived self-efficacy to cope, but not perceived stress or helplessness, with stressful experiences was vulnerable to cognitive decline, as suggested by both the group difference in PSS-PW, but not PSS-NW scores, and the significant correlation between PSS-PW, but not PSS-NW scores, and global cognition across subjects. Perceived self-efficacy often reflects underlying executive control capacity, while perceived helplessness reflects automatic threat responses [37, 38]. Self-efficacy to cope with stressors is critical for adaptively regulating stress [13]. Neurologically, perceived stress overall can affect PFC involved function and functional networks [39]. Specifically, perceived self-efficacy in control and mastery over stressors is related to the dorsal pathway of PFC, while perceived helplessness is related to the ventral pathway of PFC [40, 41]. In the context of cognitive aging, older adults tend to switch from using ventral to dorsal pathway of PFC in supporting most cognitive function [42], while dorsal pathway is more vulnerable in AD-associated neurodegeneration [43]. Synthesizing these aspects, perceived self-efficacy towards stressful experience is more relevant to cognitive function in an aging population, especially those at risk for AD.

As far as we know, this is the first study to examine the neural mechanisms underlying stress adaptation using pattern-based similarity analysis. We hypothesized that an adaptive neural recovery process would be documented by higher similarity in large-scale functional network connectivity between baseline and recovery. To examine recovery following cognitive challenges, we first identified common intrinsic resting-state networks including the DMN, aDMN, SAL, BG, CEN, FPN, SSN, VIS, and the cerebellum-midbrain network [29] in aging populations, including those at risk for AD. Current literature suggests that patterns of functional network connectivity can serve as an individual’s unique brain “fingerprint” for behavioral regulation [44, 45]. Consistent with the literature [29], some of the networks showed significantly group difference in activations in the current study. Compared to HC, aMCI showed lower activation in regions within selected networks: bilateral middle temporal lobe in the DMN, right caudate in the aDMN, superior temporal gyrus in the SAL, left amygdala in the BG, left middle frontal gyrus in the CEN, left precentral gyrus in the FPN, and left middle cingulate in the SSN. Previous studies have proposed that stress tasks may induce reorganization in large-scale brain networks including the hippocampus and PFC [15, 33, 46]. Hence, we suggest the integrity of functional network connectivity is essential for stress adaptation, and, thus, would be associated with neural recovery as an index of capacity for stress adaptation. Of note, we did not find group differences in the neural patterns of recovery. The aMCI group is known to utilize certain networks (e.g., CEN) to compensate for neural disruptions seen in the DMN and to maintain cognitive function [47, 48]. This type of compensatory mechanism should be investigated in future studies of stress adaptation and regulation in aMCI.

More global perceived self-efficacy or coping capacity in the face of stressors was associated with greater neural recovery in HC but not aMCI. Previous psychology literature lends evidence to support the protective role of positive perceptions, often reflecting more control in the context of stress, including in older adults [49, 50]. In particular, when facing stress or challenges, greater perceived self-efficacy may enhance dopamine release, which helps with neural efficiency involved in stress regulation [51]. The triggered series of neuroendocrine reactions may modulate neural excitability and plasticity and lead to widespread resource reallocation across networks [15]. Noticeably, stress adaptation is a process, consisting of multiple components (e.g., stress appraisal and acute stress recovery) and their interactions [13]. Hence, even though there were no group differences in neural recovery to cognitive challenges, the lack of association between stress-related perceptions and neural outcomes among the aMCI group suggests altered stress adaptation processes in AD-related cognitive impairment and neuropathophysiology. Altered or impaired stress adaptation can affect multiple regulatory processes that determine healthy or unhealthy aging (e.g., increases in inflammation, stress hormones, and oxidative stress) and make older adults more vulnerable to AD-associated neurodegeneration [52, 53]. Findings observed in aMCI here should be further replicated and extended, particularly longitudinally, to fully understand how altered stress regulatory processes accelerate the onset and progress of cognitive deterioration.

We need to acknowledge that the sample size for investigating the difference in neural recovery between aMCI and HC was relatively small. When comparing resting-state activation in default networks between aMCI and HC, only regions with a limited number of voxels were discovered (uncorrected *p* < 0.005). We suspect that the difference between aMCI and HC is less substantial than the difference between AD and HC. Furthermore, we only examined neural recovery to cognitive challenges; whether neural recovery would be similar across different acute challenges or stressors (e.g., physical, social, emotional) in aging populations needs to be further investigated.

Our findings, using pattern-based similarity analysis of functional network connectivity, suggest alterations in links between stress perceptions and neural adaptation in aMCI. As previously observed, cognitive vulnerability or decline appears associated with altered stress appraisal. Further study of stress adaptation in aMCI may help shed light on pathways or networks that contribute to the onset and progress of cognitive deterioration in aging.

## MATERIALS AND METHODS

### Participants

The study was approved by the University of Rochester’s Research Subject Review Board. All aMCI participants were recruited from university-affiliated memory clinics, and the diagnoses of aMCI was based on the clinical diagnosis of “mild cognitive impairment due to Alzheimer’s disease” [54]. All aMCI participants had deficits in memory (Rey’s Auditory Verbal Learning test delayed recall (RAVLT-DR) < -1SD from age-adjusted population norm), but intact basic activities of daily living. They were free of dementia based on a comprehensive neuropsychological battery and the NINCDS-ADRDA criteria per assessments. Participants had to be stable on AD medication for three months prior to enrollment. Age-, sex-, and education-matched HC participants were recruited from the community. All HC participants did not have self-reported history of dementia or aMCI and had RAVLT-DR >= age-adjusted population norm). For both groups, participants were further screened based on the following inclusion/ exclusion criteria: (1) be ≥ 60 years of age, English-speaking, and community-dwelling; (2) be able to give consent based on the research team’s assessment; (3) have adequate visual and auditory acuity for testing; (4) no severe cardiovascular disease (e.g., chronic heart failure); (5) no severe uncontrollable psychiatric disorders (e.g., major depression); (6) no severe inflammatory disease (e.g., irritable bowel syndrome); and (7) no MRI contraindications (e.g., pacemaker, claustrophobia). A total of 35 participants (17 aMCI, 18 HC) had valid data on both neuroimaging and self-reported global perceived stress.

### Experimental design

**Figure 3:**
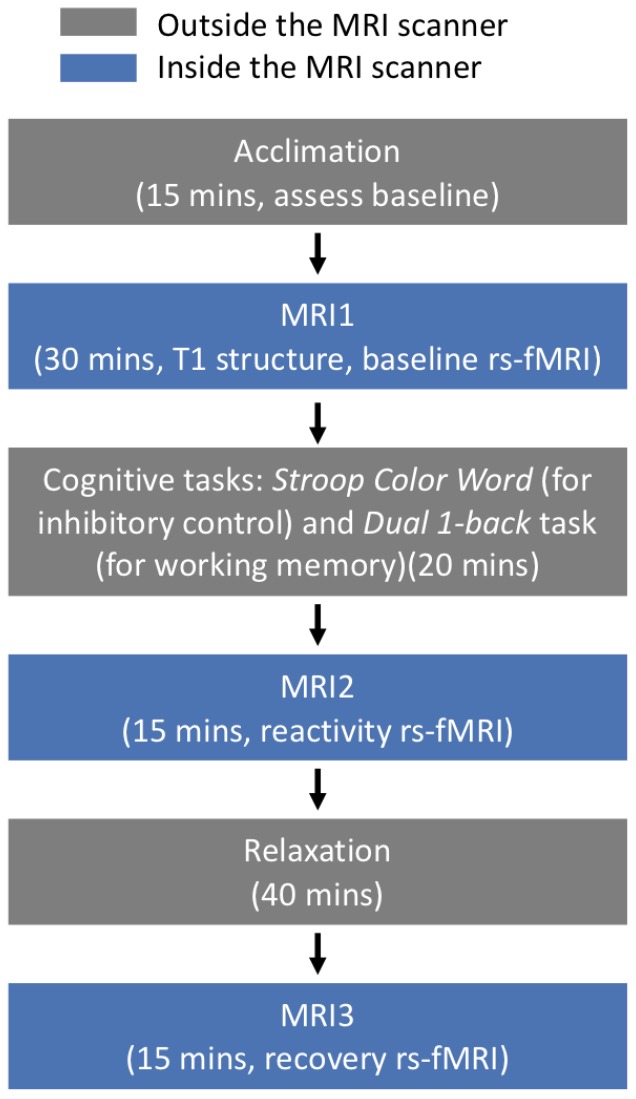
**Experimental protocol to induce acute stress.**

The study consisted of two cross-sectional sessions within a two-week window. The first session included psychological and behavioral interviews. The second session included cognitive challenge tasks and resting-state functional magnetic resonance imaging (rs-fMRI) scans. The protocol included: acclimation (15 minutes, assess baseline), MRI1 (30 minutes for T1 structure and baseline rs-fMRI), cognitive challenge tasks outside the MRI scanner (20 minutes), MRI2 (15 minutes for reactivity rs-fMRI), relaxation outside the MRI scanner (40 minutes), and MRI3 (15 minutes for recovery rs-fMRI) (Figure 3). Of note, electrocardiography data was collected at acclimation, cognitive challenge tasks, and relaxation phases. The cognitive tasks included two commonly used computerized tasks: Stroop Color Word (for inhibitory control) and Dual 1-back task (for working memory). For the Stroop task, participants were shown serial colored words on the screen and asked to judge the color of the font, regardless of the meaning of the word, as quickly and accurately as possible. For the Dual 1-back task, participants were shown an English letter on the screen and asked to judge whether the current stimulus matched the letter and position of the previous one as quickly and accurately as possible. For both tasks, feedback was displayed after participants responded to each individual trial. Reaction time and accuracy from the two tasks were recorded for further analysis. Each task lasted 10 minutes, and the order of the two tasks was randomized across participants. Instructions and practice were provided before each formal task. The group difference in performance (intra-individual difference in reaction time, IIVRT) for the two tasks are reported in Table 1.

### T1 structural MRI and BOLD rs-fMRI data acquisition

All fMRI data were collected using a 3.0 Tesla Siemens TrioTIM scanner (Erlangen, Germany) equipped with a 32-channel receive-only head and body coil transmission at the Rochester Center for Brain Imaging. Structural images obtained using an MPRAGE sequence (TR = 2530 ms, TE = 3.44 ms, TI = 1100 ms, FA = 7°, matrix = 256×256, spatial resolution = 1×1×1 mm^3^, number of slices = 192) and then used for registration during preprocessing. Rs-fMRI data were acquired with a gradient echo-planar imaging (EPI) sequence (TR = 3000 ms, TE = 30 ms, FA° = 90, matrix = 64×64, spatial resolution = 4×4×4 mm^3^, number of slices = 30, number of volumes = 100). Participants were instructed to relax while keeping their eyes open during the entire scan.

### Image preprocessing

All rs-fMRI data were preprocessed using the Data Processing Assistant for Resting-State fMRI (DPARSFA) [55], based on the Statistical Parametric Mapping software package version 8 (SPM8, http://www.fil.ion. ucl.ac.uk/spm/). Across individuals, the first five volumes were discarded to avoid potential noise related to the equilibrium of the scanner and the participants’ adaptation to the scanner. The remaining 95 volumes were preprocessed using slice time correction and motion correction. Next, the images were registered to each individual’s own structural image, normalized to the Montreal Neurological Institute (MNI) standard space (resliced to 3×3×3 mm^3^) and spatially smoothed using a Gaussian kernel (FWHM = 4 mm). Then the linear trends were removed, and a band-pass filter (0.01 - 0.08 Hz) was applied to remove long-term physiological shifts and non-neural signals.

To control for potential brain atrophy in the following analysis, VBM was performed to extract gray matter volume using the SPM8 package (http://www.fil.ion.ucl.ac.uk/spm). Briefly, the structural images were segmented into gray matter, white matter, and cerebrospinal fluid. After an initial affine registration of the gray matter map into the MNI space, the gray matter images were nonlinearly warped using diffeomorphic anatomical registration through Exponentiated Lie Algebra [56].

### Acute neural recovery from cognitive challenges

We followed the procedure of pattern-based similarity analysis utilizing rs-fMRI data described previously [57].

We first identified intrinsic neural networks from baseline rs-fMRI across all subjects using group ICA. Pattern-based similarity analysis was then applied to assess the change of functional network connectivity for each subject after acute stress tasks and a relaxation period.

### Group ICA

Was used to identify default resting-state networks. After preprocessing, we pooled rs-fMRI data from MCI and HC groups at baseline (n = 35) and performed group ICA using the GIFT toolbox [58]. During group ICA, individual data were concatenated across time to identify independent components, then subject-specific components and time series were calculated using back-reconstruction. Briefly, first, the dimensionality of each participant’s data was reduced using principal component analysis (PCA). The resulting volumes were then concatenated and the number of independent sources was estimated using the GIFT dimensionality estimation tool (n = 20, auto-selected by the estimation tool). PCA was performed again to reduce the data to the estimated dimensions (n = 20). Second, an independent component estimation was performed using the Infomax algorithm. Last, individual time series and spatial maps were computed for each participant. After back-reconstruction, the mean spatial maps were transformed to Z-values for display. Same identified ICs were applied to data at all three conditions (baseline, reactivity and recovery) in the following pattern-based similarity analysis.

### Pattern-based similarity analysis

For each participant, we extracted subject-specific time series for each component (such subject and component-specific time series was generated from group ICA) at different conditions (i.e., baseline, reactivity, and recovery). The functional network connectivity matrices were constructed by computing the Pearson correlation coefficient between all pairs of identified time series. The functional connectivity matrices for each subject were then Fisher’s Z-transformed and vectorized. Across all participants, paired *t*-tests were conducted to compare averaged functional connectivity matrices at different conditions (baseline vs. reactivity: t_(54)_ = -9.74, *p* < .001; reactivity vs. recovery: t_(54)_ = 1.29, *p* = .20). The insignificant difference between reactivity vs. recovery statuses was reasonable since some participants did not recover sufficiently to baseline status within that period of time.

**Figure 4:**
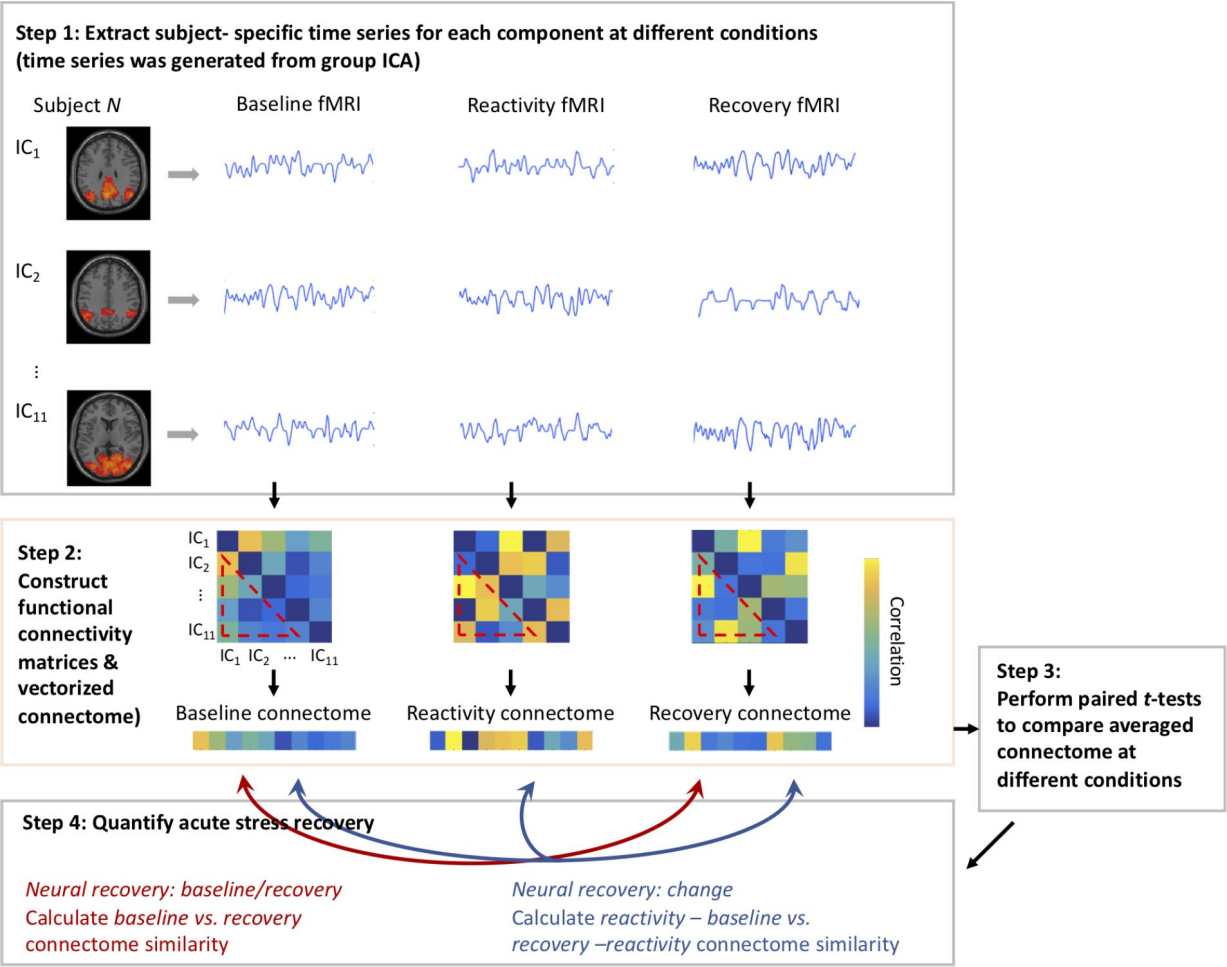
**Schematic of using pattern-based similarity analysis to assess neural recovery.**

Two approaches were used to quantify acute neural recovery from the cognitive challenges: 1) *the similarity of ICA-based networks between baseline and recovery,* indexed as *“neural recovery: baseline/recovery”,* were compared by correlating Pearson’s *r* baseline and recovery connectome vectors; and 2) *the similarity between reactivity change* (the difference in ICA-based networks between reactivity and baseline) *and recovery change* (the difference in ICA-based networks between recovery and reactivity), indexed as *“neural recovery: change”,* were compared by correlating Pearson’s *r* (reactivity - baseline) and (recovery - reactivity) connectome vectors. The purpose of adding the 2^nd^ approach was to take into account the magnitude of brain alterations induced by reactivity. A schematic of the acute neural recovery extraction is shown in Figure 4.

Of note, we reported the results from the high- frequency heart rate variability (HF-HRV) of the electrocardiography data in a separate paper [59]. Briefly, we examined the pattern of the acute neural recovery (acclimation, mean of two cognitive challenge tasks, and relaxation) with repeated measure ANOVA for the entire sample. We found that the quadratic model of HF-HRV was superior (F = 23.68, df = 1, p = .001) to the linear (F = 0.83, df = 1, p = .37) or cubic (F = 4.26, df = 1, p = .046) models, indicating the validity of the acute neural recovery.

### Measurements

### Global perceptions of stress

Were measured using the PSS [21]. The PSS questionnaire includes a total of 10 items, with the severity of perceived stress over the past month rated using a five-point scale from “never” (0) to “very often” (4). Validity of the PSS questionnaire is demonstrated in studies of older adults without and with aMCI [36, 60]. The PSS consists of 4 PSS-PW items, such as feeling confident or being in control, and 6 PSS-NW items, such as feeling nervous, upset, or angry. A two-factor model has been well-supported in the literature, with the PSS-PW subscale suggested to represent perceived self-efficacy and ability to cope, and the PSS-NW suggested to index perceived stress, distress, or helplessness [36, 61, 62]. A total of 35 participants (17 aMCI and 18 HC) with completed data were included in the following analyses. Cronbach’s alpha for the two subscales were .83 and .89, respectively, in the current study.

We also measured global cognition with the MoCA [63] and depressive symptoms with the Geriatric Depression Scale (GDS) [64].

### Statistical analysis

Statistical analysis was conducted using SPSS 22.0. Group comparisons (aMCI vs. HC) on sample characteristics, natural log transformed PSS subscales, and neural recovery measures were analyzed using independent *t*-tests for continuous variables and *χ*^2^ tests for categorical variables. Pearson’s correlation was applied to examine the relationship between PSS subscales and psychological measurements for all subjects. GLMs with an identity link and linear scale response were used to examine both the main effect of PSS subscales (or *Y* = *β_main_* × PSS subscales + *ε*) and the interaction effect of PSS subscales and group (or *Y* = *β* × PSS subscales + *β* × Group + *β_interact_* × PSS subscales × Group) on acute neural recovery. The HC group was used as the reference group for determining the interaction effect with Wald’s test in the GLMs. All tests within a two-tailed *p* < 0.05 were considered significant.

Post-hoc correlational analyses were conducted to examine the relationship between PSS subscales and neural recovery measures within each group.
